# Fast-track interventions for HIV and AIDS epidemic control among key populations: A rapid review

**DOI:** 10.4102/phcfm.v16i1.4088

**Published:** 2024-04-08

**Authors:** Zamasomi P.B. Luvuno, Ebenezer Wiafe, NomaKhosi Mpofana, Makgobole M. Urusla, Celenkosini T. Nxumalo

**Affiliations:** 1 Centre for Rural Health, School of Nursing and Public Health, University of KwaZulu-Natal, Howard Campus, Durban, South Africa; 2 Discipline of Pharmaceutical Sciences, College of Health Sciences, University of KwaZulu-Natal, Westville, Durban, South Africa; 3 Department of Somatology, Faculty of Health Sciences, Durban University of Technology, Ritson Campus, Durban, South Africa; 4 Academic Development Unit, Faculty of Health Sciences, Durban University of Technology, Durban, South Africa; 5 Discipline of Nursing, School of Nursing and Public Health, University of KwaZulu-Natal, Durban, South Africa

**Keywords:** HIV epidemic control, HIV epidemic, public health, fast track interventions, HIV/AIDS

## Abstract

**Background:**

Targeted interventions for key populations remain critical for realisation of epidemic control for human immunodeficiency virus (HIV) infection because of the causal relationship between HIV infection in the general population and among key population groups.

**Aim:**

To consolidate evidence on the fast-track interventions towards achieving HIV epidemic control among key populations.

**Methods:**

A rapid scoping review was conducted using the methodological framework by Arksey and O’ Malley. The Population, Intervention, Context and Outcome (PICO) framework was used to identify relevant studies using key words with Boolean operators in electronic data bases, namely CINHAL, Web of Science, Psych Info and Sabinet. Studies were extracted using a modified data extraction tool, and results were presented narratively.

**Results:**

A total of 19 articles were included in this review. Most articles were primary studies (*n* = 17), while another involved the review of existing literature and policies (*n* = 2) and routinely collected data (*n* = 1). Most studies were conducted in the United States of America (*n* = 6), while another were conducted in China, Kenya, Botswana, South Africa and Mozambique. All studies revealed findings on tested interventions to achieve HIV epidemic control among key populations.

**Conclusion:**

Effective interventions for HIV epidemic control were stand-alone behavioural preventive interventions, stand-alone biomedical preventive strategies and combination prevention approaches. Furthermore, the findings suggest that effective activities to achieve HIV epidemic control among key populations should be centred around prevention.

**Contribution:**

The findings of this study have policy and practice implications for high HIV burden settings such as South Africa in terms of interventions to facilitate realisation of the Joint United Nations Programme on HIV/AIDS (UNAIDS) 95-95-95 targets, thereby contributing to HIV epidemic control.

## Introduction

It is estimated that key populations, including men who have sex with men (MSM), sex workers, people who inject drugs (PWID), transgender persons, and people in prison and other closed settings and their sexual partners, account for more than half of the world’s estimated human immunodeficiency virus (HIV) infections.^1^ Individuals from key populations often lack adequate access to HIV prevention and treatment services. This subsequently results in higher rates of undiagnosed HIV, leading to higher rates of morbidity and mortality in these individuals.^2^ Key population groups accessing HIV treatment services also often experience difficulties related to viral suppression and retention into care because of issues around stigma, violence and discrimination, which impede access to health services.^3^

Epidemiological data on the global incidence and prevalence of HIV infection are attributed significantly to the estimates of incidence among key and vulnerable populations.^4^ The exact magnitude of this problem remains unclear because of historical lack of routine data on key population individuals. Moreover, there is growing evidence to suggest an overlapping network between key populations and the general population, indicating that HIV among key populations is not isolated, and therefore requires an urgent response in line with the existing preventive and treatment approaches within the HIV cascade.^5^ Research, globally, shows that key populations are disproportionality affected by HIV/AIDS (acquired immune deficiency syndrome) when compared to the general population.^6^ Furthermore, there are also reports of multiple sexual partners with low condom usage in most regions around the world.^7^ This subsequently results in higher rates of other sexually transmitted infections (STIs), which precipitate a long-term continuance of such infections.

There have been multiple programmatic calls to refocus prevention, treatment and care initiatives so as to comprehensively target individuals from key population groups through biomedical and preventive interventions to achieve HIV epidemic control.^8^ The foundational impediments to HIV epidemic control among key populations are accurate data on the size of these populations, their location, profile of risk behaviours per region and country, and the exact ratio of key population versus general population HIV incidence and prevalence. To achieve the Joint United Nations Programme on HIV/AIDS (UNAIDS) 95-95-95 fast-track targets, the health system must address issues related to the prevention, treatment, and care of the unique needs of key populations.^9^ Addressing the needs of key populations requires intensified programming, targeted resources allocation, and contextually relevant and responsive interventions that address and counteract the systematic marginalisation and discrimination often experienced by key populations.

The UNAIDS 95-95-95 targets on HIV/AIDS provide a road map for directing interventions to facilitate the implementation of fast-track interventions to achieve HIV epidemic control, so that HIV/AIDS is no longer a public health threat by 2030. While several interventions have been instituted with specific focus on impact strategies to halt the spread of new infections, many regions are yet to achieve the UNAIDS 95-95-95 targets. Targeted interventions for key population groups remain a key driver to facilitating realisation of epidemic control for HIV infection. The purpose of this review is thus to consolidate evidence on the fast-track interventions towards achieving HIV epidemic control among key populations. For the purpose of this review, HIV epidemic control refers to interventions or strategies to facilitate realisation of the UNAIDS 95-95-95 targets towards eliminating HIV/AIDS as a major public health threat. The UNAIDS 95-95-95 targets aim to facilitate epidemic control through fast-track interventions that call for 95% of individuals to know their HIV status through HIV testing, 95% of people to be initiated and retained on HIV treatment, and 95% to be virologically suppressed while on antiretrovirals.^10^

## Methods

This rapid systematic scoping review maps evidence of fast-track interventions to achieve HIV/AIDS epidemic control among key population groups. The methodological framework by Arksey and O’Malley^11^ was used to guide the review. The following steps were subsequently followed when carrying out the review: (1) identification of the review question, (2) identification of relevant studies, (3) selection of relevant studies, (4) charting of the data and (5) collating, summarising, and reporting the findings.

### Identifying the research question

The main research question was ‘What is the existing evidence on fast-track interventions to achieve HIV/AIDS epidemic control among key populations?’

The research sub-questions are:

1. What evidence exists on the recommendations and practices to achieve HIV/AIDS epidemic control among key populations?
2. What is the range of literature on the current best practices for facilitating the implementation of the fast track the UNAIDS 95-95-95 HIV/AIDS treatment cascade for key populations?
3. Which practices and implemented recommendations have been effective?

### Identification of relevant studies

This study used the Population, Intervention, Context and Outcome (PICO) framework to align the study selection with the research question (see Table 1).

**TABLE 1:** Population, Intervention, Context and Outcome framework.

Population	Intervention	Context	Outcome
Key population individuals	Recommendations or practices for HIV epidemic control	HIV infection	HIV epidemic control

HIV, human immunodeficiency virus.

### Search strategy

A combination of relevant search terms with Boolean operators were used to search for published literature in the following databases: CINAHL, Web of Science, Psych Info and Sabinet. Grey literature and other databases were not searched because of the rapid nature of the review which sought to provide evidence-based information to inform researchers and policy makers on empirically tested interventions for key populations to achieve HIV epidemic control within the context of the UNAIDS 95-95-95 strategy. Because of the evolving landscape of HIV/AIDS research within key population, the rapid review sought to provide timely evidence to inform policy makers on relevant interventions that may inform clinical practice. Table 2 provides a description of the search words used and how search words were combined to obtain relevant articles.

**TABLE 2:** Details of search terms used to obtain articles from databases.

SN	Search terms used
#1	ALL= (“Recommendations” OR “Practices” OR “Guidelines”)
#2	ALL= (“Achieve” OR “Attain”)
#3	ALL= (“HIV” OR “AIDS” OR “Human Immunodeficiency virus” OR “Acquired Immunodeficiency syndrome”)
#4	ALL= (“Epidemic Control” OR “Reduction” OR “Prevention” OR “Control”)
#5	ALL= (“Key populations” OR “men who have sex with men” OR “Sex workers” OR “Drug addicts” OR “Transgender persons” OR “Prisoners” OR “Sexual partners” OR “Vulnerable populations” OR “Sexual minority”)
#6	#1 AND #2
#7	#3 AND #4
#8	#6 AND #7
#9	#8 AND #5

### Selection of eligible studies

Four reviewers conducted the literature search and uploaded all literature search results on Endnote X20 software. A thorough title and abstract screening was then carried out by all the four authors, and duplicates removal was facilitated at this stage. Relevant articles for full text review were then selected, and all the four reviewers independently conducted screening of articles to ensure that they met inclusion criteria for full text reviews. A fifth independent reviewer was subsequently employed to ensure that all articles included for full text review aligned to the inclusion criteria and also facilitated consensus in terms of thematic description of results that emerged from the review process that was conducted by the initial four authors. The following inclusion and exclusion criteria were applied to identify relevant studies:

#### Inclusion criteria

- Studies from the year 2014 onwards as this was the year that the UNAIDS 90-90-90 strategy was adopted.
- English published studies.
- Studies that included HIV fast-track interventions for key populations with intervention outcomes.

#### Exclusion criteria

- Research articles that are not primary empirical studies that present interventions and outcomes related to interventions to achieve HIV epidemic control among key populations.
- Grey literature, opinion, articles, commentaries, policy documents and dissertations.
- Studies published prior to the year 2014.

### Charting the data

**FIGURE 1:**
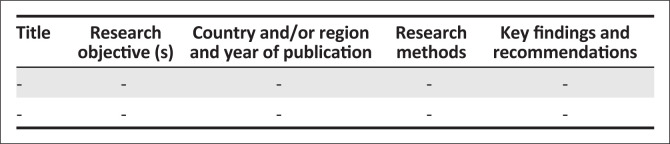
Data charting template.

A data charting form was used to electronically capture relevant data from the studies included in the review. Figure 1 provides details of the electronic data capturing tool that was used to chart the studies included in this review.

### Collating, summarising and reporting the findings

A narrative report was formulated to provide a summary of the data extracted based on the elements of the data charting template. Emerging results were discussed thematically, in relation to the objectives of the scoping review, and in line with the PICO framework which was used to identify the relevant studies. The results are subsequently discussed in the context of broader existing literature on the interventions to facilitate HIV epidemic control among key and vulnerable populations.

### Ethical considerations

This article followed all ethical standards for research without direct contact with human or animal subjects.

## Review findings

**FIGURE 2:**
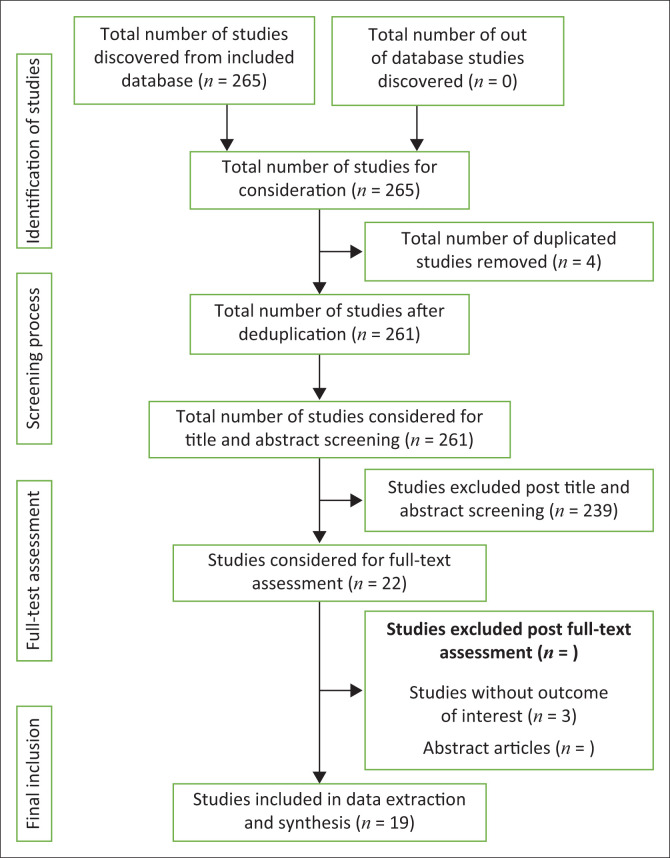
Summary of study selection process.

The initial search yielded 265 articles and no further searches were conducted thereafter. Figure 2 illustrates the search and selection process. A total of 19 articles were eventually included for this review. Appendix Table 1-A1 provides an overview of the articles and the key findings using the following headings: author, title, research objectives, country and/or region and year of publication, research methods, key findings and recommendations. Most articles included in this review were primary studies (*n* = 17), while others involved the review of existing literature and policies (*n* = 1) and routinely collected data (*n* = 1). Of the 19 articles included, 12 employed quantitative methods (*n* = 12),^12,13,14,15,16,17,18,19,20,21,22,23^ 5 used qualitative approaches (*n* = 5)^24,25,26,27,28^; only one study (*n* = 1)^29^ employed mixed methods approaches while yet another study comprised a review of literature and policy documents collectively (*n* = 1).^30^ Most studies (*n* = 6) were conducted in the United States of America,^12,13,17,22,26,28^ while others were conducted in China (*n* = 5),^14,15,16,21,23^ Kenya (*n* = 2),^18,24^ South Africa (*n* = 1),^20^ Mozambique (*n* = 1),^29^ Nigeria (*n* = 1),^25^ Botswana (*n* = 1)^27^ and New Guinea (*n* = 1).^19^ The remaining studies were unaccounted for in terms of country and region, as these were document reviews conducted on regional policies and research. Since the main aim of this review was mapping of evidence related to fast-track interventions to achieve HIV/AIDS epidemic control among key populations, quality appraisal of articles reviewed was not conducted.

Broadly speaking, the articles reviewed suggest that recommendations to achieve HIV epidemic control among key populations are centred on stand-alone biomedical and behavioural interventions and combination approaches, incorporating both medical and psychosocial approaches. Based on the articles reviewed, most studies reported on interventions directed towards MSM (*n* = 11).^12,13,16,17,18,20,23,25,26,28^ The remaining studies reported on recommendations and interventions directed towards female sex workers (*n* = 7),^14,15,19,24,27,29,30^ and drug users (*n* = 1).^21^ It was interesting to note that there were no studies reporting on the transgender population.

## Discussion

The aim of this scoping review was to map evidence of existing fast-track interventions to achieve HIV epidemic control among key populations. The findings of this review suggest that the effective interventions for HIV epidemic control were stand-alone behavioural preventive interventions, stand-alone biomedical preventive strategies and combination prevention approaches. Furthermore, the findings of this review imply that effective activities to achieve HIV epidemic control among key populations should be centred around preventive activities.

In the MSM population, it was found that key interventions to facilitate HIV epidemic control using behaviour modification encompassed education and health promotions approaches around the use of HIV preventive methods such as condoms, water-based lubricants and promoting HIV testing. Research on the epidemiology of HIV infection among MSM advocates that prevention strategies be formulated to prevent escalating incidences of HIV infection among this key population sub-group.^31,32^ The findings of an early meta-analytic review of behaviour interventions to prevent HIV risk behaviours among MSM suggested that interventions be based on sexual risk reduction and incorporate theoretical models to include elements of interpersonal skills training, with delivery spanning multiple sessions and different delivery modalities.^33^

Johnson et al.^34^ also found that behavioural interventions to reduce risk of infection were effective interventions for HIV prevention among MSM. Typically, these strategies include reduction of unprotected sex through individualised counselling on protection against HIV and other STIs, and provision of behavioural support through peer education, relationship support, and discussions on the attitudes and beliefs around HIV preventive methods.^34^ On the other hand, Lorimer et al.^35^ found that group and community-level behavioural interventions for MSM are effective; however, expanding the scope of routine data indicators is required for monitoring the impact of specific behavioural interventions that are effective among MSM. Alluding to behavioural interventions to facilitate HIV prevention among MSM, Safren et al.^36^ state that such interventions must also address the mental and psychosocial challenges that are faced by MSM, as these have an influence on sexual health behaviours that often exacerbate the risk for HIV infection.

Other behaviour modification strategies of epidemic control addressed issues of treatment compliance in men who were already HIV positive. Human immunodeficiency virus treatment as an HIV preventive strategy is another intervention that has been recommended, as supported by the universal test and treat strategy; provision of treatment adherence support was found to be an effective intervention in this regard. Study findings show that the use of innovative modalities incorporating social media and e-health approaches yields positive results in terms of fostering and maintaining adherence support, particularly among MSM youth. Hergenrather et al.^37^ further postulate that behavioural interventions for HIV prevention among MSM should incorporate the needs of the diverse, well-educated and web-based millennial generation, and differentiate between adolescents and young adults.

For female sex workers, previous research studies show that behavioural interventions are effective in reducing HIV infection and incidences of other STIs. In this regard, social support as an intervention was found to positively correlate with the adoption of HIV prevention approaches such as condom use; more specifically, social support in the form of informational and emotional support was associated with HIV prevention. Female sex workers, co-workers and partners were instrumental in this regard. The application of cognitive behavioural theories in the promotion of condom usage and uptake yielded positive outcomes in terms of HIV risk reduction. These findings have also been shown to have similar implications for HIV reduction among people injecting drugs.^38,39,40^ While behavioural interventions have been found to be effective in promoting HIV preventive behaviours such as condom use and HIV testing,^41^ maximising effectiveness requires sustainability which necessitates ongoing investment from government and related funding agencies.^42^

Since stand-alone behavioural interventions are shown to be an effective strategy for HIV epidemic control among key populations, especially MSM, female sex workers and PWID, it is essential that these intervention strategies be tailored to individual needs, while also addressing the structural and systemic issues that hinder access to health service, taking cognisance of the multiple influences of access to health service at all levels in society. Since the findings of the review also suggest that stand-alone biomedical HIV preventive interventions such as Pre-exposure Prophylaxis (PrEP), non-Occupational Post Exposure Prophylaxis (Npep), condom usage and voluntary medical male circumcision are also effective approaches to achieving HIV epidemic control among MSM, female sex workers and PWID, maintaining the uptake of these services is crucial for sustained epidemic control. Research of these biomedical HIV prevention approaches has shown that understanding and addressing the contextual driver and barriers is important for maximum uptake. In the context of key population individuals, sustained uptake is also dependent on an understanding of the unique health needs of individuals who form part of key populations. Research studies on access to health services for key populations have also advocated for policy changes to facilitate inclusion of the unique needs of these individuals. Moreover, this has prompted the need for education and training of health professionals, both preservice and in-service.

In terms of biomedical HIV preventive strategies, it was found that facilitating different modelling approaches to scale up PrEP yielded tangible outcomes in terms of a reduction in new rates of infection among MSM. Early initiation of antiretroviral therapy (ART) was found to be a predictor for viral suppression in order to halt the further spread of infection. Similarly, the findings of a mathematical model of biomedical interventions for HIV prevention among MSM, in China, suggested that PrEP test and treatment, and their combinations are cost-effective and operative approaches to HIV prevention among MSM in the context of China’s HIV response strategy.^43^ Similar studies conducted in other low-income settings also provide evidence of the effectiveness of combined PrEP and early ART initiation as preventive approaches to facilitating HIV prevention among MSM. While evidence on the aforementioned notion of combined prevention is compelling,^31,44^ there is a need for interventions to be instituted to address the contextual social and structural issues that hinder uptake, and the accessibility of combined biomedical HIV preventive methods. Creating an awareness of the specific contextual psychosocial structural and systemic factors that affect MSM thus becomes imperative, and should therefore be the entry point for the creation of relevant interventions.^45,46,47^ Present literature in this regard relates to issues of stigma, discrimination, racism, individual perceptual factors, community norms, values, religion, societal practices and other social injustices.^45,47,48,49,50,51^ These are key barriers hindering the uptake of these combined biomedical approaches and thus have implications for broader public health.

Facilitating the availability of nPEP was also found to be an effective strategy for HIV prevention among MSM. An early study on nPEP as a bio-behavioural HIV prevention intervention suggested that while nPEP appeared to be feasible and safe when used at community level in groups, successful integration of this strategy into existing comprehensive HIV prevention programmes was unclear.^52^ On the other hand, observational data recommends the adoption of nPEP to reduce the likelihood of HIV infection following potential exposure.^53^ The maximum benefits of this strategy are, however, challenged by poor uptake related to acceptability and a lack of knowledge regarding this strategy, particularly among same gender loving men and the MSM population.^53,54,55^

Provision of PrEP as a biomedical preventive strategy was also effective among female sex workers. Observational data has also demonstrated the effectiveness of PrEP for HIV prevention, more particularly, for key populations such as female sex workers who are at greater risk of HIV infection.^56,57^ Similar to the MSM population, understanding the contextual drivers and barriers to uptake thus becomes essential for the yield of maximum benefits of this preventive approach. In this regard, understanding issues of acceptability and other psychosocial factors that are barriers to accessibility and uptake of PrEP, is vital especially among female sex workers.^58,59,60^ The provision of voluntary medical male circumcision was found to be an effective biomedical preventive strategy for reducing the incidence of HIV infection among female sex workers. This finding has significant implications for public health in terms of the creation of a demand for voluntary medical male circumcision, and further concurs with previous findings on the effectiveness of medical circumcision as a biomedical HIV prevention strategy.^61,62,63^

Specific interventions for HIV epidemic control for PWID are centred on biomedical preventive interventions promoting harm reduction through the provision of safe opioid substitution therapy, and facilitating the availability of needle and syringes for drug users. The needle and syringe programme reduces the chances of person-to-person spread of HIV infection by preventing drug needle sharing. This also simultaneously prevents the spread of other communicable diseases. Other studies have shown that behavioural intervention with combined harm reduction and biomedical approaches such as the provision of PrEP for PWID, are more effective for HIV prevention.^64,65,66^ Success of this intervention is, however, challenged by the multiplicity of factors that hinder continued access to PrEP among PWID.^67^ These factors also relate to stigma and structural issues related to legislation around PWID, particularly in low-to-middle income settings such as sub-Saharan Africa.^68^ Contextual interventions for HIV prevention among PWID should therefore include targeted interventions addressing systemic and structural issues, so that tangible outputs may be attained and sustained for existing interventions.^69^

Combination HIV prevention strategies for HIV epidemic control entailed the use of both behavioural and biomedical approaches, and were found to be specifically effective among MSM and female sex workers. For MSM, the studies reviewed showed that men who had been exposed to combined preventive approaches were more likely to consistently practise safe sexual intercourse by using water-based lubricants, condoms and testing regularly for HIV. For female sex workers, study findings showed that combination approaches, rooted in the provision of social support systems for sex workers, yield effective results in terms of compliance with the HIV preventive strategies such as consistent condom use and HIV testing. Specific strategies, such as the promotion of self-efficacy and HIV self-testing, were found to be acceptable and effective methods of facilitating HIV prevention, thereby resulting in epidemic control. Moreover, self-efficacy was found to be a predictor of consistent condom use among female sex workers for both stable and casual partners. There was also evidence to suggest that combination approaches targeted at female sex workers address structural issues related to legislation and health policy to prevent poor health-seeking behaviours. Specific interventions in this regard were related to the creation of sex worker-friendly facilities and cooperation between sex workers and government authorities in the design of policies for health service delivery to address the health needs of sex workers.

Broadly speaking, research advocates for combined approaches to HIV prevention among key populations.^43,70,71,72^ The effectiveness of combined approaches, taking on individualised and community-based approaches encompassing behaviour and psychosocial elements, is also widely cited and suggested.^73,74,75^ The concept of individualised approaches brings to light the notion of person-centred holistic and comprehensive care,^76^ while the community-based approach acknowledges the role of community values, norms and attributes and how they shape health behaviour.^77^ Collectively, the aforementioned issues may account for the core social determinants of health that influence health behaviour and, ultimately, health-seeking practices. This should thus be the foundation of all interventions instituted with the aim of achieving HIV epidemic control among key populations. Combined biomedical and behavioural approaches necessitate comprehensive and integrated approaches ensuring adoption and maintenance of HIV preventive methods to facilitate HIV epidemic control. This necessitates skilled health professionals to integrate multiple behavioural, psychosocial and biomedical theories and practices in the process of providing care within the recommended policy frameworks. Training of health professionals on the multiple realities of the health system and diverse health needs of individuals, especially key populations, thus becomes a crucial strategy that is required from a clinical practice perspective, health policy perspective, and from health science education perspective.

The review conducted presents major implications for clinical practice in relation to HIV epidemic control in high HIV burden regions such as South Africa, as it has provided information on the targeted and effective interventions for HIV epidemic control among key populations. The findings are based on the synthesis of evidence that is provided by the mainly primary data which demonstrates interventions and their outcomes in terms of HIV epidemic control. While the findings of the review are instrumental in guiding policy and practice interventions, certain key gaps in interventions and research are noted in this area.

It is interesting to note that, based on this review, there were no effective interventions found which targeted the transgender population. Moreover, gaps exist in the number of targeted interventions directed towards the treatment, care and retention cascade of the HIV response strategy. Other gaps as highlighted by the review related to specific effective interventions for adolescent and young adult key population groups. Interventions and related research are therefore necessary in this area. On the basis of the review results, a summary of recommendations for health policy, practice, education and research is proposed next and may be applied in high burden HIV settings like South Africa.

## Recommendations for health policy related to key populations

- Comprehensive guidelines should be provided pertaining to the clinical management guidelines for care and support for key population individuals, with individualised and comprehensive service packages for each sub-group. These guidelines should not be a one-size-fits-all, but should rather be contextualised in terms of the various levels of the health system and health care. The guidelines should further accommodate the different roles and categories of healthcare workers in the multidisciplinary health team commencing with community level.
- A communication strategy for facilitating the uptake of various behavioural, biomedical and combination preventive approaches must be developed and disseminated, taking cognisance of specific religious, cultural, community and societal values and norms, and should transcend language barriers and other communication impediments such as physical and cognitive disability.
- Development of a population estimate for all subgroups of key population individuals with geographical hotspot mapping to facilitate implementation and monitoring of targeted interventions for epidemic control should ensue. This should be accompanied by the development of clearly defined statistical indicators to measure performance of health facilities in terms of activities to achieve HIV epidemic control among different key population subgroups across the HIV response cascade.
- The development of an e-health strategy should be enabled for the roll-out of behavioural, biomedical and combination HIV prevention approaches, using telemedicine, social media and M-health approaches for key populations, particularly targeting adolescents and young adults. These should be contextualised, based on literacy levels and access to technology and other digital health devices.

## Recommendations for clinical practice and health service delivery related to key populations

- Scale up behavioural, biomedical and combination prevention approaches for key populations at community level, targeting key influences of clients.
- Facilitate intersectoral and multidisciplinary partnerships for largescale community-based health promotion interventions aimed at achieving HIV epidemic control among key populations.
- Health facilities to facilitate ‘train-the-trainer’ interventions directed towards education of communities on key populations, in order to address the social barriers hindering the uptake of HIV preventive interventions among key populations. Community-led behaviour change interventions should be implemented to facilitate HIV preventive behaviours among key populations.
- Creation of key population-friendly health services at health facilities, building on the existing approaches for adolescent- and youth-friendly services.
- Interventions targeting adolescent and young key populations should incorporate elements of edutainment and fast-tracked service delivery to promote retention and continued accessibility of health services.
- Improve accessibility of biomedical HIV preventive services such as PrEP and nPEP to extend beyond fixed health clinics to include community-based sites such as mobile clinic points, treatment pick-up points, and hotspot areas where key populations are often found.
- Outreach services to facilitate scale-up of biomedical preventive approaches to extend beyond the normal daytime hours into the night, to accommodate key populations such as PWID and sex workers. Mobile outreach services for HIV preventive and treatment services should also extend after-hours into the night to facilitate improved access to services.
- HIV epidemic control interventions should extend beyond harm reduction activities and should also include an emphasis on HIV prevention, treatment and care through combined behavioural and biomedical approaches.

## Recommendations for future research and education

- All categories of health professionals should be trained on the clinical care and holistic supportive management of key population individuals. Health science education curricula should thus include this content in their undergraduate and post-graduate health science qualifications. Moreover, higher education institutions should highlight issues of key population health and HIV focal research in undergraduate and post-graduate research. This will increase the body of research literature available concerning this study area, especially in South Africa and sub-Saharan Africa, where there is a continued dearth of primary empirical research in this area.
- This review demonstrated that research and effective interventions for transgender population are presently minimal, and that primary research, incorporating diverse research approaches and designs is necessary among this population.
- More primary research among the adolescent and young adult key population groups is required, with focus on the health needs of this population, with mathematical modelling of the impact of proposed and implemented interventions and evaluation studies on the actual outcomes of targeted interventions. This is particularly necessary for low-to-middle income settings such as South Africa and sub-Saharan Africa.
- Research should also be particularly directed towards PWID with the focus on health needs of this population and the potential outcomes of combined harm reduction and HIV preventive services.

## Limitations

While this review provides evidence that has important public health and policy implications related to effective strategies for HIV epidemic control, especially for high burden HIV settings like South Africa, it is not without limitations. These relate to the nature of this systematic scoping review which was rapid in nature, with a focus on studies that had documented outcomes of effective interventions that were measured quantitatively, qualitatively and through modelling approaches. This review was also further limited by the fact that only three scholarly databases were consulted, and articles chosen were from 2014 onward, with only English articles being selected. While the findings of the review provide evidence to inform practice, findings are limited because of the aforementioned limitations. Nonetheless, the findings highlight important interventions to be considered and point to possible gaps to be addressed by policy, practice and research in the quest to achieve HIV epidemic control among key populations. The findings and related implications are additionally important for high HIV burden settings like South Africa, where the HIV response strategy is committed to achieving HIV epidemic control with a special focus on key populations.

## Conclusion

The findings of this review revealed that the key effective strategies for achieving HIV epidemic control among key populations centre on stand-alone, behavioural and biomedical interventions, and combination prevention approaches. In this regard, the findings imply that achieving HIV epidemic control among key populations should centre on prevention strategies. The findings do, however, show gaps in research and effective interventions targeting transgender individuals and people injecting drugs. The review further highlights the lack of effective interventions related to the treatment and care cascade. Lastly, the findings of this review also suggest that achieving HIV epidemic control among key populations is reliant on combined strategies that simultaneously address the structural, systemic and psychosocial barriers hindering access to healthcare.

## Appendix 1

**TABLE 1-A1:** Summary of research studies on effective practices for human immunodeficiency virus epidemic control among key populations.

#	Author	Title	Research objectives	Country/region and year	Research methods	Key findings	Recommendations
1.	Firestone et al.	Effectiveness of a combination prevention strategy for HIV risk reduction in men who have sex with men in Central America: A mid-term evaluation	To assess the effectiveness of a combination prevention programme for reducing HIV risk among MSM across 5 countries in Central America	Central America 2014	Data were collected from a sample of 3531 men using a behavioural survey. Routine programme data were also used to ascertain programme coverage with 15 031 programme contacts being identified. Coarsened Exact Matching (CEM) and Spearman correlation coefficients were used to assess the relationship between potential matching variables and any programme exposure	Men exposed to both behavioural and biomedical components related to HIV prevention were more likely to use condoms and water-based lubricants at the last sexual encounter, while those exposed to behavioural interventions were more likely to have tested for HIV in the past year	Combination prevention programmes are promising; however, expansion in coverage and intensity of programmes is required using multi-levelled approaches
2.	LeVasseur et al.	The effect of PrEP on HIV incidence among men who have sex with men in the context of condom use, treatment as prevention and seroadaptive practices	To evaluate the impact of increasing PrEP uptake in conjunction with established prevention strategies on HIV incidence in a high-risk population of MSM through simulation	USA 2018	An agent-based simulation model representing the sexual behaviour of high risk, urban MSM in the USA over a period of 1 year was used to evaluate the effect of PrEP on HIV infection rates	Simulation modelling suggests that PrEP uptake at 25% per 10 000 MSM prevented 30.7% of infections as a stand-alone preventive strategy. In the absence of PrEP, HIV treatment as prevention, condom use and sero-adaptive behaviours independently, prevented 27.1%, 48.8% and 37.7% infections respectively. The addition of PrEP to the aforementioned preventive methods, prevented an additional 5% of infections	Multipronged approaches incorporating access to PrEP, HIV testing, access to ART promotion of condom usage must be used to facilitate HIV infection reduction among people who engage in high risk sexual activity
3.	Qiao et al.	Social support and condom use among female sex workers in China	To explore the association between social support from various sources and condom use with clients and stable partners	China 2014	A cross-sectional survey among female sex workers from two different cities with high HIV prevalence. Data were collected from a sample of 1022 participants. Data were analysed using analysis of variance, bivariate and multivariate logistic regression analysis to explore the relationship between demographic variables and different forms of social support in relation to condom usage	Co-workers were the main source of informational support for sex workers, while friends were the leading sources of emotional support. Stable partners were found to be a tangible source of support for female sex workers Emotional support was positively associated with correct condom use, while informational support correlated with consistent condom use with stable partners. Overall, the quantity of social support received by sex workers was low	Creation of a positive environment for condom possession and use by sex workers through specific provisions facilitated by policy changes that address structural barriers such as criminalisation, alleviating stigma and provision of modern methods of accessing condoms in areas accessible to sex workers
4.	Stapele, Nencel & Sabelis	On tensions and opportunities: Building partnerships between government and sex worker-led organisations in Kenya in the Fight Against HIV/AIDS	To analyse the obstacles, enabling conditions and moments of dialogue between government organisations and sex-worker-led organisations in developing horizontal partnerships in HIV/AIDS prevention and care, to contribute to their political inclusion	Kenya 2019	A qualitative approach using semi-structured interview methods to collect data from 20 government officials, NGOs and sex worker activists	The findings suggest that horizontal partnerships between sex workers and government officials is a crucial step to achieving a more unified and effective response to HIV/AIDS	Fostering community participation in relation to HIV prevention among sex workers is integral to facilitating horizontal partnerships
5.	Zhang et al.	Predictors of consistent condom use among Chinese female sex workers: An application of the Protection Motivation Theory	To assess the predictors of intention and behaviour of consistent condom use among Chinese female sex workers	China 2014	A cross-sectional survey using a self-administered questionnaire among 700 female sex workers was used to collect data. Pearson Product Moment Correlations and multivariate logistic regression analysis were used to assess and examine the association between theoretical framework constructs and consistent condom use and behaviour intention	Higher levels of intention for consistent condom use were prevalent for casual sexual partners, rather than for stable sexual partners. The findings also suggest that self-efficacy is a predictor for consistent condom use with both stable and casual sexual partners	HIV prevention interventions should devise targeted interventions to reduce sex workers’ perceptions of reward for inconsistent condom use, reduce perceptions of response cost for using a condom, and promote condom use self-efficacy through enhancing communication skills of sex workers and provision of training on emotional control with both stable and casual sexual partners
6.	Lafort et al.	Feasibility, acceptability and potential sustainability of a diagonal approach to health services for female sex workers in Mozambique	To test a diagonal approach to health service delivery	Mozambique 2018	The study applied a convergent parallel mixed method design, combining analysis of the implementation process with semi-structured interviews from key informants, structured interviews with health facility contact persons, and focus group discussions with peer outreach workers. The methodological framework for health systems and Consolidated framework for implementation science were used to collectively guide the study	The diagonal intervention, encompassing targeted interventions expanded to sex workers and creating friendlier sex worker services was found not to be feasible because of insufficient political will and lack of resources for targeted components of the intervention	Making public health services sex worker-friendly has been proven effective, however, strong peer outreach and community mobilisation is an essential component, together with availability of long-term funding for similar initiatives
7.	Liu et al.	Predictors of antiretroviral initiation: a cross-sectional study among Chinese HIV infected men who have sex with men	To assess the predictors of ART initiation among Chinese HIV infected MSM	China 2015	A cross-sectional survey among HIV infected MSM with use of logistic regression analysis to evaluate sociodemographic factors and behavioural factors associated with ART initiation	Older age and having adverse HIV clinical symptoms were associated with ART initiation. Moreover, married MSM were more likely to be initiated and remain on ART, as opposed to those who were unmarried	The findings suggest that built-in educational and psychological interventions would be necessary to cultivate a sense of responsibility among MSM and their spouses, so as to facilitate early ART initiation
8.	Kuhn et al.	Adaption of evidence-based approaches to promote HIV testing and treatment engagement among high-risk Nigerian youth	To inform adaptation of an evidence-based peer navigation and health approaches for combination interventions to promote HIV testing and care engagement among youth MSM in Nigeria	Nigeria 2021	Semi-structured interviews through focus group discussions were used to collect data from youth MSM, community stakeholders, academic experts and governmental sectors	The findings suggest that personalisation of messages, use of local social media platforms such as WhatsApp, Grindr and Facebook were effective in reaching youth to promote HIV testing and care engagement among youth MSM	Scaled up intervention roll-out and testing is required to determine the outcomes of this intervention. Issues of safety, sustainability and training of peer mentors with use of this approach is also necessary
9.	LeGrand et al.	Epic Allies: development of a gaming App to improve Antiretroviral Therapy adherence among young HIV positive Men who have sex with men	To report on the process in creating an ART adherence app for young MSM	USA 2016	A three-phase development process was followed which included: (1) theory-based development, (2) assessment of target populations ART adherence and (3) development and usability testing	Results confirmed appropriateness of a game-based app within social networking features to address issues of ART adherence among young MSM	Upscaling and testing of commercial HIV apps that are grounded in theoretical foundations is required, so as to identify sources of information and facilitate motivation, behaviour change and medication adherence
10.	Arnold et al.	Identifying social and economic barriers to regular care and treatment for black Men who have sex with men, and women and who are living with HIV: a qualitative study from Bruthas cohort	To explore the treatment and care experiences of HIV positive black Men who have sex with men	USA 2017	Behavioural survey underpinned by a randomised control trial was used to facilitate an understanding of the social and economic barriers to treatment care among MSM, within a behavioural intervention for sexual health	Individual level intervention focusing on behavioural risk reduction for HIV transmission provided promising results in terms of reduction of HIV risk behaviour. However, there were problems noted in accessing and limitations in social security which undermined access to healthcare services and HIV care	Tailored interventions for HIV positive black MSM should address the structural barriers affecting remaining care and must include group level activities that increase social support for these men in terms of finances and multi-level stigma. There should also be a deliberate attempt to improve treatment literacy
11.	Kunzweiler et al.	Factors associated with viral suppression among HIV-positive Kenyan gay and bisexual MSM	To identify factors associated with viral suppression among HIV-positive gay and bisexual MSM who were offered treatment in a supportive programme	Kenya 2018	A longitudinal cohort study among gay and bisexual men who have sex with men who were recruited through snowball sampling from an existing network of 200 men	Early initiation of ART was a predictor for viral suppression; however, men who reported receptive or versatile sexual position during anal intercourse with a male partner, had reduced odds of viral suppression. Greater levels of coping efficacy were associated with increased odds of viral suppression	Coping self-efficacy represents a crucial promotive factor of resilience for viral suppression and must thus be facilitated at an individual level upon ART initiation and through the treatment cascade
12.	Shava et al.	Acceptability of oral HIV self-testing among female sex workers in Gaborone, Botswana	To assess acceptability of, obstacles to and preferred approaches to HIV self-testing among female sex workers in Gaborone, Botswana	Botswana 2020	Semi-structured interviews were conducted with female sex workers, nurses and lay counsellors	Oral HIV self-testing was found to be a highly acceptable method of HIV testing among female sex workers	Implementation of HIV self-testing should be peer-driven with healthcare worker oversight. Implementation of HIV self-testing should also identify and address structural barriers in the health system that may hinder roll-out and uptake of this intervention. Multi-disciplinary approaches should be employed to address knowledge gaps
13.	Wu et al.	Implementation of HIV non-occupational post-exposure prophylaxis for men who have sex with men in two cities of Southwestern China	To describe the efficacy of non-occupational HIV post-exposure prophylaxis among MSM in China	Southwestern China (2021)	The study enrolled MSM individuals prescribed on non-occupation post-exposure prophylaxis and evaluated efficacy of intervention based on follow retention, adherence and HIV sero-conversion	Implementation of non-occupational exposure prophylaxis is an imperative strategy for HIV prevention among MSM. Results of the study showed no sero-conversions at 4- and 6-week intervals. Only one recipient was found to convert at 3 and 6 months respectively	Non-occupational post-exposure prophylaxis should be an integrated HIV prevention strategy used with PrEP with provision of individualised peer support to promote adherence
14.	Beyer et al.	An action agenda for HIV and sex work	To present synthesised evidence as advocacy to address HIV among sex workers	Global 2015	Systematic review and evidence synthesis	Literature reviewed reveals that models of simulation suggest that condom promotion could reduce HIV prevalence by 70% among sex-workers and their partners, while voluntary access to PrEP could reduce HIV incidence by 40%	Preventive interventions for sex workers must be delivered as a comprehensive package that is community based and sex-worker led. Moreover, interventions must be cross-cutting to reach different sub-populations of sex workers
15.	Gray et al.	Impact of male circumcision on the HIV epidemic in Papua New Guinea: A country with extensive foreskin cutting practices	To estimate the likely impact of medical male circumcision on HIV incidence reduction	New Guinea 2014	An age-structured mathematical model was used, including rural and urban populations, and the impact of STI prevalence was examined as a co-factor in HIV transmission and acquisition	In the presence of medical male circumcision, there were marked increases in condom use among female sex workers and their partners, and reduction in HIV incidence by 10%	Combination HIV prevention, including VMMC for men, must be continuously upscaled among the general population
16.	Fortenberry, Koenig & Kapogiannis	Implementation of an Integrated Approach to the National HIV/AIDS Strategy for Improving Human Immunodeficiency Virus Care for Youths	To summarise a national, multi-agency and multi-level approach to HIV care for newly diagnosed youths	USA 2017	Three federal agencies developed memoranda of understanding to sequentially implement three protocols addressing key National HIV/AIDS Strategy goals. The goals were addressed in the Adolescent Trials Network, with protocols implemented in 12 to 15 sites across the USA	A total of 3986 HIV-positive youths were referred for care, with more than 75% linked to care within 6 weeks of referral, with almost 90% of those youths engaged in subsequent HIV care. Community mobilisation efforts implemented and completed structural change objectives to address local barriers to care. Age and racial/ethnic group disparities were addressed through targeted training for culturally competent, youth-friendly care, and intensive motivational interviewing training.	A differentiated model of care for HIV is required for youths with services that can be comprehensively packaged in a manner that is age-appropriate considering contextual issues of stigma and the dynamics associated with being a youth
17.	Freese et al.	Real-World Strategies to engage and retain racial-ethnic minority young men who have sex with men in HIV prevention services	To present findings from a national summit on racial/ethnic young MSM services	USA 2017	Sub-group discussions focused on issues related to treatment access, outreach/engagement/retention, continuing care/recovery support and health literacy for the minority. The nominal Group Technique was used to identify the strategies and approaches that summit participants believed to be most promising for engaging and retaining minority YMSM in HIV prevention services	Analyses of results from summit activities highlight four key cross-cutting strategies – utilising peers, providing holistic care, making services fun, and utilising technology – as critical for engaging minority YMSM in HIV prevention care	Understanding contextual factors that comprehensively promote uptake of HIV preventive initiatives is crucial to service delivery among these key populations
18.	William et al.	Intervening to identify and reduce drug use and sexual HIV risk patterns among men who have sex with men in three provinces in South Africa	To report findings of an intervention programme to address drug use and reduce sexual risk behaviour among MSM	South Africa 2014	Retrospective review of routinely collected data from intervention	Targeted interventions for drug using MSM populations reached 3475 of these individuals as facilitated by non-governmental organisations. Communication outreach promoting HIV prevention and addressing drug abuse was most effective with HIV testing and referral being the most prominent target attainable	Addressing unsafe drug usage and drug abuse should be an essential component of the HIV treatment and prevention cascade among MSM
19.	Benedikt et al.	Allocative and implementation efficiency of HIV prevention and treatment for people who inject drugs	To present strategies and outcomes of interventions to promote HIV prevention among people injecting drugs	Eastern Europe and central Asia 2016	Allocative and implementation efficiency analysis methods were applied. *Optima*, a dynamic, population-based HIV model with an integrated programme and economic analysis framework was applied	Implementation of needle and syringe programme, provision of opioid substitution therapy for treatment of drug dependence, with integration of HIV prevention services through testing and referral within these programmes. There is evidence to suggest that 26% of new infections will be averted through such intervention while 34% of related deaths will be prevented	Development of a combined HIV testing and treatment intervention within syringe programmes, clear targets and monitoring systems to be facilitated

MSM, men who have sex with men; STI, sexually transmitted infection; USA, United States of America; NGOs, non-governmental organisations; HIV, human immunodeficiency virus; AIDS, acquired immune deficiency syndrome; ART, antiretroviral therapy; VMMC, Voluntary Medical Male Circumcision; YMSM, Young Men who have Sex with Men.
